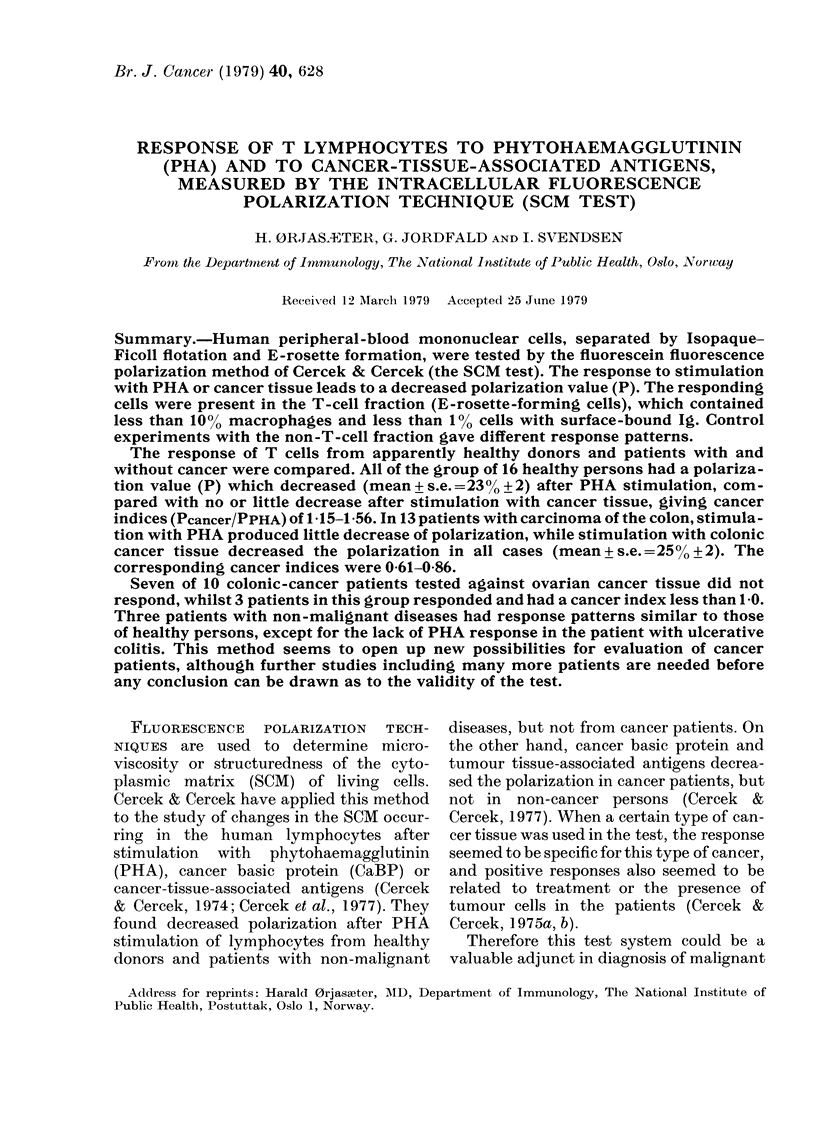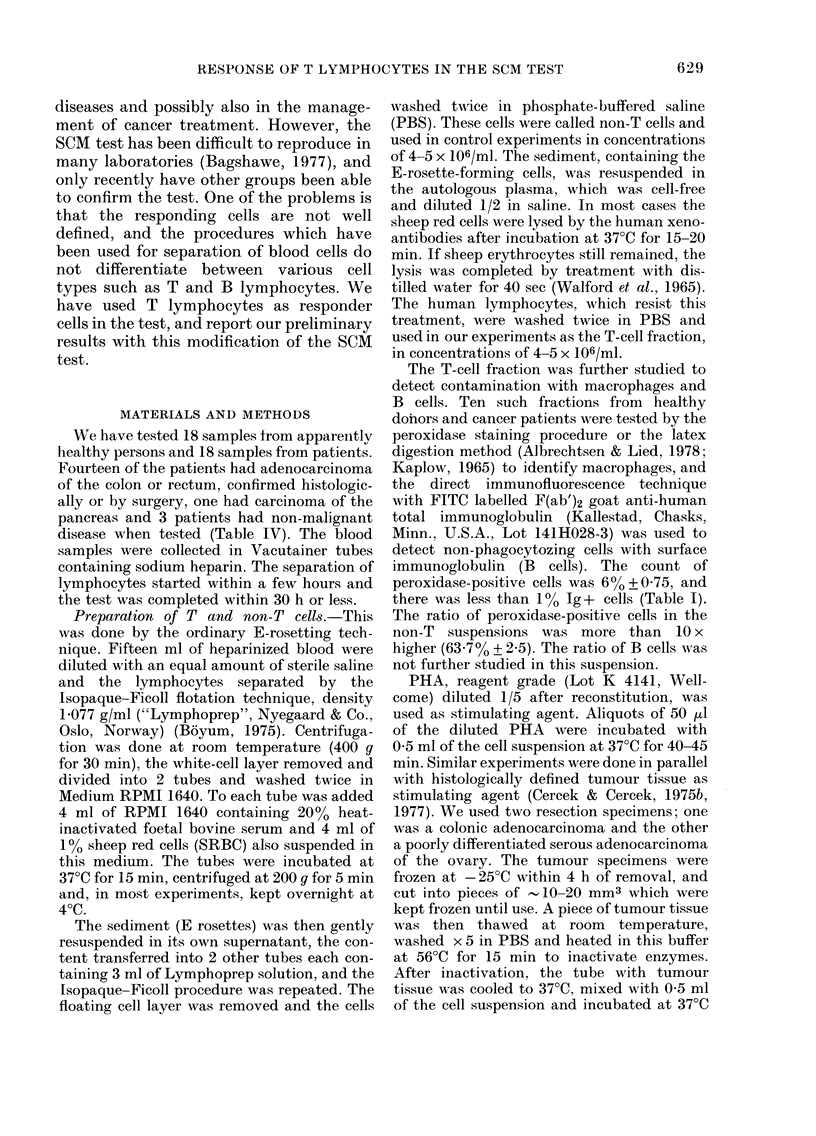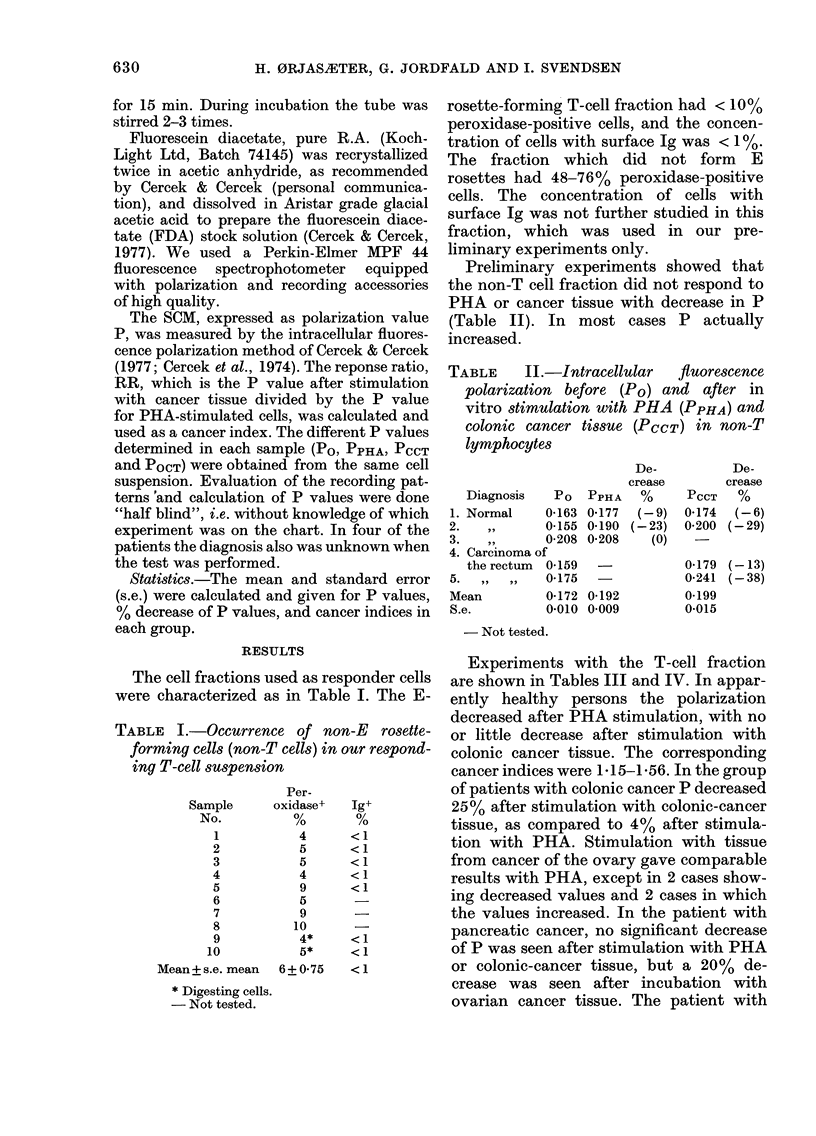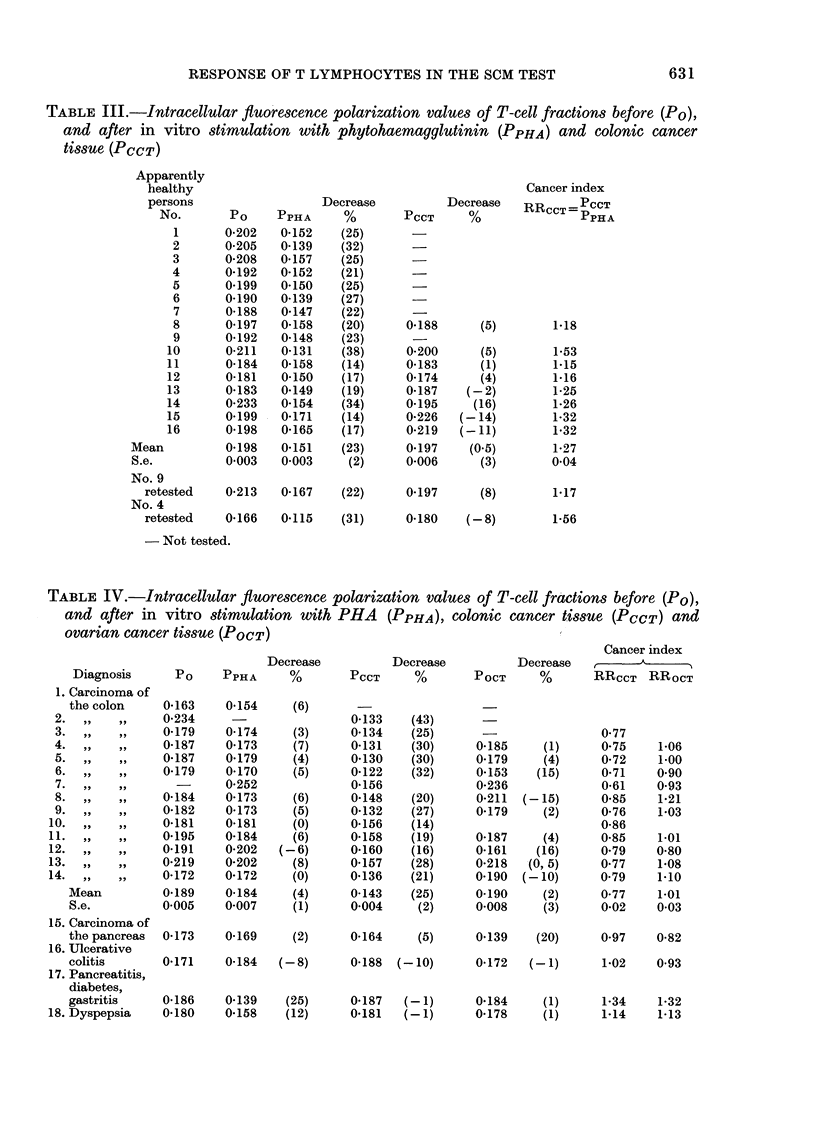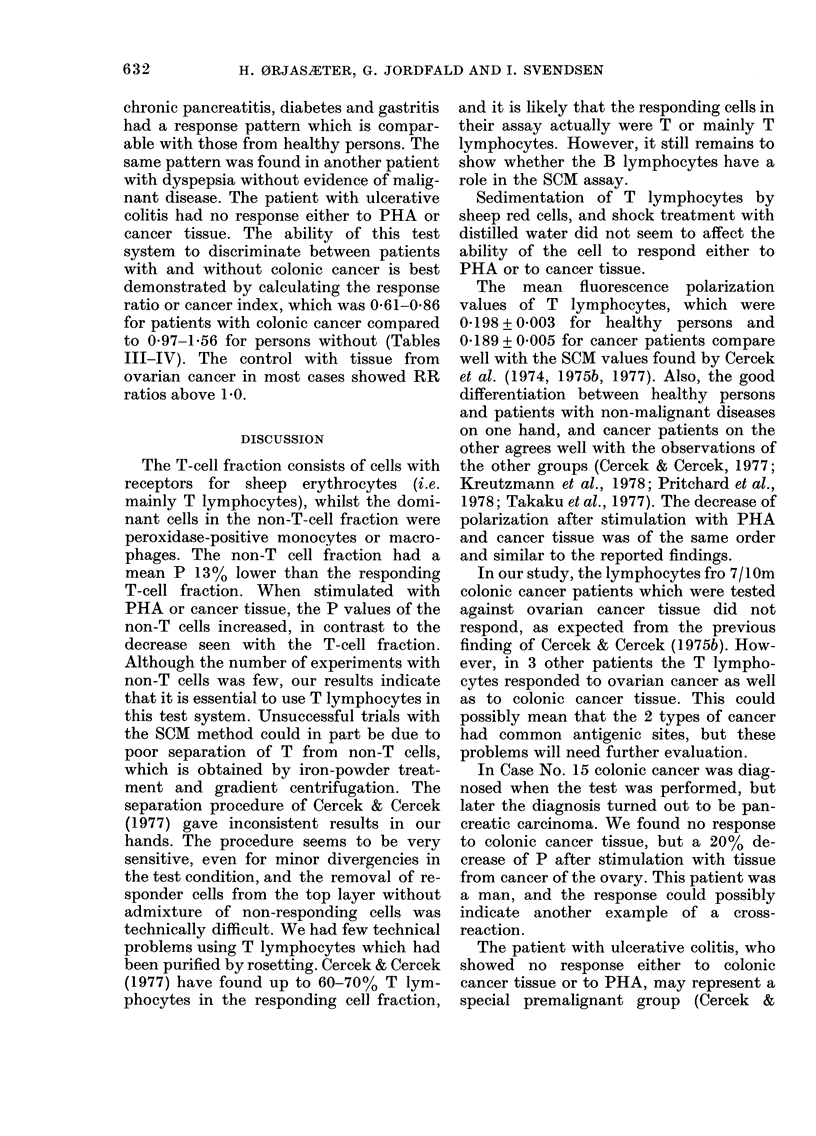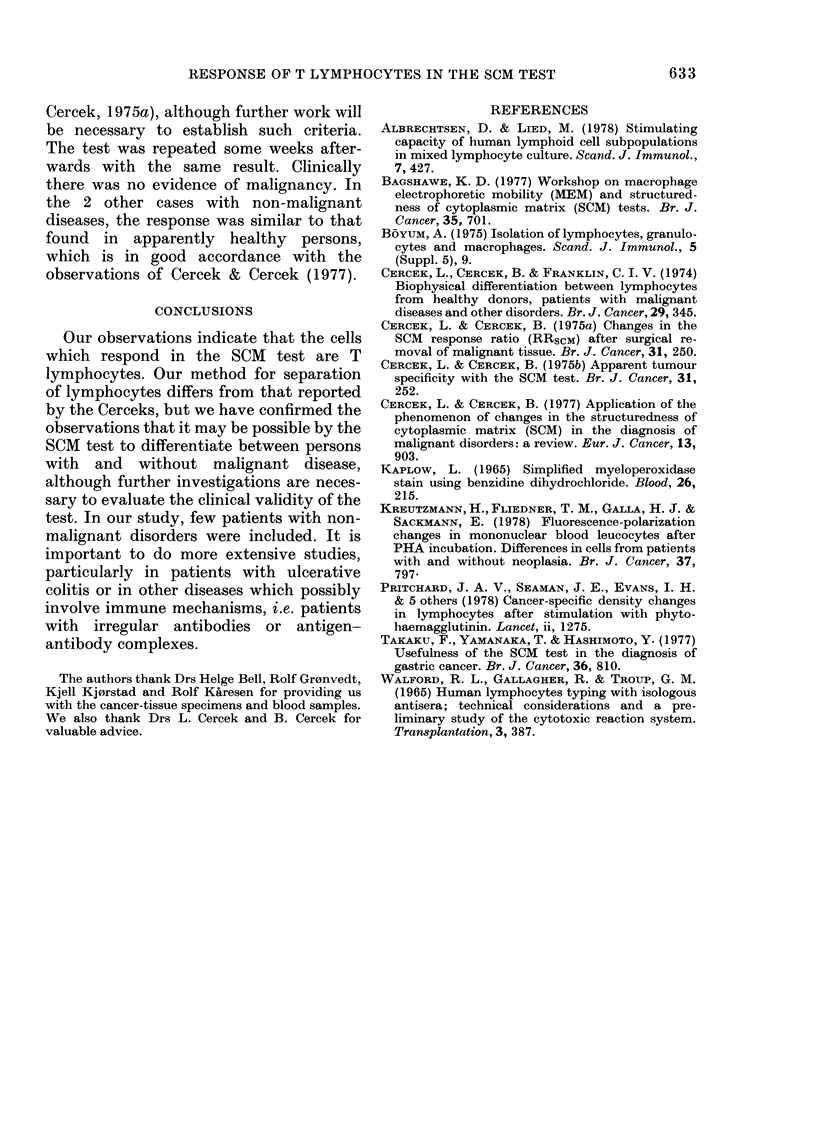# Response of T lymphocytes to phytohaemagglutinin (PHA) and to cancer-tissue-associated antigens, measured by the intracellular fluorescence polarization technique (SCM test).

**DOI:** 10.1038/bjc.1979.228

**Published:** 1979-10

**Authors:** H. Orjasaeter, G. Jordfald, I. Svendsen

## Abstract

Human peripheral-blood mononuclear cells, separated by Isopaque-Ficoll flotation and E-rosette formation, were tested by the fluorescein fluorescence polarization method of Cercek & Cercek (the SCM test). The response to stimulation with PHA or cancer tissue leads to a decreased polarization value TP). The responding cells were present in the T-cell fraction (E-rosette-forming cells), which contained less than 10% macrophages and less than 1% cells with surface-bound Ig. Control experiments with the non-T-cell fraction gave different response patterns. The response of T cells from apparently healthy donors and patients with and without cancer were compared. All of the group of 16 healthy persons had a polarization value (P) which decreased (mean +/- s.e. = 23% +/- 2) after PHA stimulation, compared with no or little decrease after stimulation with cancer tissue, giving cancer indices (P cancer/PPHA) of 1.15--1.56. In 13 patients with carcinoma of the colon, stimulation with PHA produced little decrease of polarization, while stimulation with colonic cancer tissue decreased the polarization in all cases (mean +/- s.e. = 25% +/- 2). The corresponding cancer indices were 0.61--0.86. Seven of 10 colonic-cancer patients tested against ovarian cancer tissue did not respond, whilst 3 patients in this group responded and had a cancer index less than 1.0. Three patients with non-malignant diseases had response patterns similar to those of healthy persons, except for the lack of PHA response in the patient with ulcerative colitis. This method seems to open up new possibilities for evaluation of cancer patients, although further studies including many more patients are needed before any conclusion can be drawn as to the validity of the test.


*Br. J. Cancer* (1979) **40**, 628

### RESPONSE OF T LYMPHOCYTES TO PHYTOHAEMAGGLUTININ (PHA) AND TO CANCER-TISSUE-ASSOCIATED ANTIGENS, MEASURED BY THE INTRACELLULAR FLUORESCENCE POLARIZATION TECHNIQUE (SCM TEST)

H. ØRJASÆTER, G. JORDFALD AND I. SVENDSEN

*From the Department of Immunology, The National Institute of Public Health, Oslo, Norway*

Received 12 March 1979 Accepted 25 June 1979

**Summary.—Human peripheral-blood mononuclear cells, separated by Isopaque–Ficoll flotation and E-rosette formation, were tested by the fluorescein fluorescence polarization method of Cercek & Cercek (the SCM test). The response to stimulation with PHA or cancer tissue leads to a decreased polarization value (P). The responding cells were present in the T-cell fraction (E-rosette-forming cells), which contained less than 10% macrophages and less than 1% cells with surface-bound Ig. Control experiments with the non-T-cell fraction gave different response patterns.**

**The response of T cells from apparently healthy donors and patients with and without cancer were compared. All of the group of 16 healthy persons had a polarization value (P) which decreased (mean ± s.e. = 23% ± 2) after PHA stimulation, compared with no or little decrease after stimulation with cancer tissue, giving cancer indices ($P_{cancer}/P_{PHA}$) of 1·15–1·56. In 13 patients with carcinoma of the colon, stimulation with PHA produced little decrease of polarization, while stimulation with colonic cancer tissue decreased the polarization in all cases (mean ± s.e. = 25% ± 2). The corresponding cancer indices were 0·61–0·86.**

**Seven of 10 colonic-cancer patients tested against ovarian cancer tissue did not respond, whilst 3 patients in this group responded and had a cancer index less than 1·0. Three patients with non-malignant diseases had response patterns similar to those of healthy persons, except for the lack of PHA response in the patient with ulcerative colitis. This method seems to open up new possibilities for evaluation of cancer patients, although further studies including many more patients are needed before any conclusion can be drawn as to the validity of the test.**

FLUORESCENCE POLARIZATION TECHNIQUES are used to determine microviscosity or structuredness of the cytoplasmic matrix (SCM) of living cells. Cercek & Cercek have applied this method to the study of changes in the SCM occurring in the human lymphocytes after stimulation with phytohaemagglutinin (PHA), cancer basic protein (CaBP) or cancer-tissue-associated antigens (Cercek & Cercek, 1974; Cercek *et al.*, 1977). They found decreased polarization after PHA stimulation of lymphocytes from healthy donors and patients with non-malignant diseases, but not from cancer patients. On the other hand, cancer basic protein and tumour tissue-associated antigens decreased the polarization in cancer patients, but not in non-cancer persons (Cercek & Cercek, 1977). When a certain type of cancer tissue was used in the test, the response seemed to be specific for this type of cancer, and positive responses also seemed to be related to treatment or the presence of tumour cells in the patients (Cercek & Cercek, 1975*a*, *b*).

Therefore this test system could be a valuable adjunct in diagnosis of malignant

Address for reprints: Harald Ørjasæter, MD, Department of Immunology, The National Institute of Public Health, Postuttak, Oslo 1, Norway.

diseases and possibly also in the management of cancer treatment. However, the SCM test has been difficult to reproduce in many laboratories (Bagshawe, 1977), and only recently have other groups been able to confirm the test. One of the problems is that the responding cells are not well defined, and the procedures which have been used for separation of blood cells do not differentiate between various cell types such as T and B lymphocytes. We have used T lymphocytes as responder cells in the test, and report our preliminary results with this modification of the SCM test.

### MATERIALS AND METHODS

We have tested 18 samples from apparently healthy persons and 18 samples from patients. Fourteen of the patients had adenocarcinoma of the colon or rectum, confirmed histologically or by surgery, one had carcinoma of the pancreas and 3 patients had non-malignant disease when tested (Table IV). The blood samples were collected in Vacutainer tubes containing sodium heparin. The separation of lymphocytes started within a few hours and the test was completed within 30 h or less.

*Preparation of T and non-T cells.*—This was done by the ordinary E-rosetting technique. Fifteen ml of heparinized blood were diluted with an equal amount of sterile saline and the lymphocytes separated by the Isopaque–Ficoll flotation technique, density 1·077 g/ml ("Lymphoprep", Nyegaard & Co., Oslo, Norway) (Böyum, 1975). Centrifugation was done at room temperature (400 *g* for 30 min), the white-cell layer removed and divided into 2 tubes and washed twice in Medium RPMI 1640. To each tube was added 4 ml of RPMI 1640 containing 20% heat-inactivated foetal bovine serum and 4 ml of 1% sheep red cells (SRBC) also suspended in this medium. The tubes were incubated at 37°C for 15 min, centrifuged at 200 *g* for 5 min and, in most experiments, kept overnight at 4°C.

The sediment (E rosettes) was then gently resuspended in its own supernatant, the content transferred into 2 other tubes each containing 3 ml of Lymphoprep solution, and the Isopaque–Ficoll procedure was repeated. The floating cell layer was removed and the cells washed twice in phosphate-buffered saline (PBS). These cells were called non-T cells and used in control experiments in concentrations of $4–5 \times 10^6$/ml. The sediment, containing the E-rosette-forming cells, was resuspended in the autologous plasma, which was cell-free and diluted 1/2 in saline. In most cases the sheep red cells were lysed by the human xeno-antibodies after incubation at 37°C for 15–20 min. If sheep erythrocytes still remained, the lysis was completed by treatment with distilled water for 40 sec (Walford *et al.*, 1965). The human lymphocytes, which resist this treatment, were washed twice in PBS and used in our experiments as the T-cell fraction, in concentrations of $4–5 \times 10^6$/ml.

The T-cell fraction was further studied to detect contamination with macrophages and B cells. Ten such fractions from healthy donors and cancer patients were tested by the peroxidase staining procedure or the latex digestion method (Albrechtsen & Lied, 1978; Kaplow, 1965) to identify macrophages, and the direct immunofluorescence technique with FITC labelled $F(ab')_2$ goat anti-human total immunoglobulin (Kallestad, Chasks, Minn., U.S.A., Lot 141H028-3) was used to detect non-phagocytozing cells with surface immunoglobulin (B cells). The count of peroxidase-positive cells was $6\% \pm 0{\cdot}75$, and there was less than 1% Ig+ cells (Table I). The ratio of peroxidase-positive cells in the non-T suspensions was more than 10× higher ($63{\cdot}7\% \pm 2{\cdot}5$). The ratio of B cells was not further studied in this suspension.

PHA, reagent grade (Lot K 4141, Wellcome) diluted 1/5 after reconstitution, was used as stimulating agent. Aliquots of 50 μl of the diluted PHA were incubated with 0·5 ml of the cell suspension at 37°C for 40–45 min. Similar experiments were done in parallel with histologically defined tumour tissue as stimulating agent (Cercek & Cercek, 1975*b*, 1977). We used two resection specimens; one was a colonic adenocarcinoma and the other a poorly differentiated serous adenocarcinoma of the ovary. The tumour specimens were frozen at −25°C within 4 h of removal, and cut into pieces of ~10–20 $mm^3$ which were kept frozen until use. A piece of tumour tissue was then thawed at room temperature, washed ×5 in PBS and heated in this buffer at 56°C for 15 min to inactivate enzymes. After inactivation, the tube with tumour tissue was cooled to 37°C, mixed with 0·5 ml of the cell suspension and incubated at 37°C

for 15 min. During incubation the tube was stirred 2–3 times.

Fluorescein diacetate, pure R.A. (Koch-Light Ltd, Batch 74145) was recrystallized twice in acetic anhydride, as recommended by Cercek & Cercek (personal communication), and dissolved in Aristar grade glacial acetic acid to prepare the fluorescein diacetate (FDA) stock solution (Cercek & Cercek, 1977). We used a Perkin-Elmer MPF 44 fluorescence spectrophotometer equipped with polarization and recording accessories of high quality.

The SCM, expressed as polarization value P, was measured by the intracellular fluorescence polarization method of Cercek & Cercek (1977; Cercek *et al.*, 1974). The reponse ratio, RR, which is the P value after stimulation with cancer tissue divided by the P value for PHA-stimulated cells, was calculated and used as a cancer index. The different P values determined in each sample ($P_O$, $P_{PHA}$, $P_{CCT}$ and $P_{OCT}$) were obtained from the same cell suspension. Evaluation of the recording patterns and calculation of P values were done "half blind", *i.e.* without knowledge of which experiment was on the chart. In four of the patients the diagnosis also was unknown when the test was performed.

*Statistics.*—The mean and standard error (s.e.) were calculated and given for P values, % decrease of P values, and cancer indices in each group.

### RESULTS

The cell fractions used as responder cells were characterized as in Table I. The E-rosette-forming T-cell fraction had < 10% peroxidase-positive cells, and the concentration of cells with surface Ig was < 1%. The fraction which did not form E rosettes had 48–76% peroxidase-positive cells. The concentration of cells with surface Ig was not further studied in this fraction, which was used in our preliminary experiments only.

TABLE I.—*Occurrence of non-E rosette-forming cells (non-T cells) in our responding T-cell suspension*

| Sample No. | Per-oxidase$^+$ % | Ig$^+$ % |
|---|---|---|
| 1 | 4 | < 1 |
| 2 | 5 | < 1 |
| 3 | 5 | < 1 |
| 4 | 4 | < 1 |
| 5 | 9 | < 1 |
| 6 | 5 | — |
| 7 | 9 | — |
| 8 | 10 | — |
| 9 | 4* | < 1 |
| 10 | 5* | < 1 |
| Mean ± s.e. mean | 6 ± 0·75 | < 1 |

* Digesting cells.
— Not tested.

Preliminary experiments showed that the non-T cell fraction did not respond to PHA or cancer tissue with decrease in P (Table II). In most cases P actually increased.

TABLE II.—*Intracellular fluorescence polarization before ($P_O$) and after in* vitro *stimulation with PHA ($P_{PHA}$) and colonic cancer tissue ($P_{CCT}$) in non-T lymphocytes*

| Diagnosis | $P_O$ | $P_{PHA}$ | Decrease % | $P_{CCT}$ | Decrease % |
|---|---|---|---|---|---|
| 1. Normal | 0·163 | 0·177 | (−9) | 0·174 | (−6) |
| 2. ” | 0·155 | 0·190 | (−23) | 0·200 | (−29) |
| 3. ” | 0·208 | 0·208 | (0) | — | |
| 4. Carcinoma of the rectum | 0·159 | — | | 0·179 | (−13) |
| 5. ” ” | 0·175 | — | | 0·241 | (−38) |
| Mean | 0·172 | 0·192 | | 0·199 | |
| S.e. | 0·010 | 0·009 | | 0·015 | |

— Not tested.

Experiments with the T-cell fraction are shown in Tables III and IV. In apparently healthy persons the polarization decreased after PHA stimulation, with no or little decrease after stimulation with colonic cancer tissue. The corresponding cancer indices were 1·15–1·56. In the group of patients with colonic cancer P decreased 25% after stimulation with colonic-cancer tissue, as compared to 4% after stimulation with PHA. Stimulation with tissue from cancer of the ovary gave comparable results with PHA, except in 2 cases showing decreased values and 2 cases in which the values increased. In the patient with pancreatic cancer, no significant decrease of P was seen after stimulation with PHA or colonic-cancer tissue, but a 20% decrease was seen after incubation with ovarian cancer tissue. The patient with

TABLE III.—*Intracellular fluorescence polarization values of T-cell fractions before* ($P_O$), *and after* in vitro *stimulation with phytohaemagglutinin* ($P_{PHA}$) *and colonic cancer tissue* ($P_{CCT}$)

| Apparently healthy persons No. | $P_O$ | $P_{PHA}$ | Decrease % | $P_{CCT}$ | Decrease % | Cancer index $RR_{CCT} = \frac{P_{CCT}}{P_{PHA}}$ |
|---|---|---|---|---|---|---|
| 1 | 0·202 | 0·152 | (25) | — | | |
| 2 | 0·205 | 0·139 | (32) | — | | |
| 3 | 0·208 | 0·157 | (25) | — | | |
| 4 | 0·192 | 0·152 | (21) | — | | |
| 5 | 0·199 | 0·150 | (25) | — | | |
| 6 | 0·190 | 0·139 | (27) | — | | |
| 7 | 0·188 | 0·147 | (22) | — | | |
| 8 | 0·197 | 0·158 | (20) | 0·188 | (5) | 1·18 |
| 9 | 0·192 | 0·148 | (23) | — | | |
| 10 | 0·211 | 0·131 | (38) | 0·200 | (5) | 1·53 |
| 11 | 0·184 | 0·158 | (14) | 0·183 | (1) | 1·15 |
| 12 | 0·181 | 0·150 | (17) | 0·174 | (4) | 1·16 |
| 13 | 0·183 | 0·149 | (19) | 0·187 | (−2) | 1·25 |
| 14 | 0·233 | 0·154 | (34) | 0·195 | (16) | 1·26 |
| 15 | 0·199 | 0·171 | (14) | 0·226 | (−14) | 1·32 |
| 16 | 0·198 | 0·165 | (17) | 0·219 | (−11) | 1·32 |
| Mean | 0·198 | 0·151 | (23) | 0·197 | (0·5) | 1·27 |
| S.e. | 0·003 | 0·003 | (2) | 0·006 | (3) | 0·04 |
| No. 9 retested | 0·213 | 0·167 | (22) | 0·197 | (8) | 1·17 |
| No. 4 retested | 0·166 | 0·115 | (31) | 0·180 | (−8) | 1·56 |

— Not tested.

TABLE IV.—*Intracellular fluorescence polarization values of T-cell fractions before* ($P_O$), *and after* in vitro *stimulation with PHA* ($P_{PHA}$), *colonic cancer tissue* ($P_{CCT}$) *and ovarian cancer tissue* ($P_{OCT}$)

| Diagnosis | $P_O$ | $P_{PHA}$ | Decrease % | $P_{CCT}$ | Decrease % | $P_{OCT}$ | Decrease % | Cancer index $RR_{CCT}$ | Cancer index $RR_{OCT}$ |
|---|---|---|---|---|---|---|---|---|---|
| 1. Carcinoma of the colon | 0·163 | 0·154 | (6) | — | | — | | | |
| 2. ,, ,, | 0·234 | — | | 0·133 | (43) | — | | | |
| 3. ,, ,, | 0·179 | 0·174 | (3) | 0·134 | (25) | — | | 0·77 | |
| 4. ,, ,, | 0·187 | 0·173 | (7) | 0·131 | (30) | 0·185 | (1) | 0·75 | 1·06 |
| 5. ,, ,, | 0·187 | 0·179 | (4) | 0·130 | (30) | 0·179 | (4) | 0·72 | 1·00 |
| 6. ,, ,, | 0·179 | 0·170 | (5) | 0·122 | (32) | 0·153 | (15) | 0·71 | 0·90 |
| 7. ,, ,, | — | 0·252 | | 0·156 | | 0·236 | | 0·61 | 0·93 |
| 8. ,, ,, | 0·184 | 0·173 | (6) | 0·148 | (20) | 0·211 | (−15) | 0·85 | 1·21 |
| 9. ,, ,, | 0·182 | 0·173 | (5) | 0·132 | (27) | 0·179 | (2) | 0·76 | 1·03 |
| 10. ,, ,, | 0·181 | 0·181 | (0) | 0·156 | (14) | | | 0·86 | |
| 11. ,, ,, | 0·195 | 0·184 | (6) | 0·158 | (19) | 0·187 | (4) | 0·85 | 1·01 |
| 12. ,, ,, | 0·191 | 0·202 | (−6) | 0·160 | (16) | 0·161 | (16) | 0·79 | 0·80 |
| 13. ,, ,, | 0·219 | 0·202 | (8) | 0·157 | (28) | 0·218 | (0, 5) | 0·77 | 1·08 |
| 14. ,, ,, | 0·172 | 0·172 | (0) | 0·136 | (21) | 0·190 | (−10) | 0·79 | 1·10 |
| Mean | 0·189 | 0·184 | (4) | 0·143 | (25) | 0·190 | (2) | 0·77 | 1·01 |
| S.e. | 0·005 | 0·007 | (1) | 0·004 | (2) | 0·008 | (3) | 0·02 | 0·03 |
| 15. Carcinoma of the pancreas | 0·173 | 0·169 | (2) | 0·164 | (5) | 0·139 | (20) | 0·97 | 0·82 |
| 16. Ulcerative colitis | 0·171 | 0·184 | (−8) | 0·188 | (−10) | 0·172 | (−1) | 1·02 | 0·93 |
| 17. Pancreatitis, diabetes, gastritis | 0·186 | 0·139 | (25) | 0·187 | (−1) | 0·184 | (1) | 1·34 | 1·32 |
| 18. Dyspepsia | 0·180 | 0·158 | (12) | 0·181 | (−1) | 0·178 | (1) | 1·14 | 1·13 |

chronic pancreatitis, diabetes and gastritis had a response pattern which is comparable with those from healthy persons. The same pattern was found in another patient with dyspepsia without evidence of malignant disease. The patient with ulcerative colitis had no response either to PHA or cancer tissue. The ability of this test system to discriminate between patients with and without colonic cancer is best demonstrated by calculating the response ratio or cancer index, which was 0·61–0·86 for patients with colonic cancer compared to 0·97–1·56 for persons without (Tables III–IV). The control with tissue from ovarian cancer in most cases showed RR ratios above 1·0.

### DISCUSSION

The T-cell fraction consists of cells with receptors for sheep erythrocytes (*i.e.* mainly T lymphocytes), whilst the dominant cells in the non-T-cell fraction were peroxidase-positive monocytes or macrophages. The non-T cell fraction had a mean P 13% lower than the responding T-cell fraction. When stimulated with PHA or cancer tissue, the P values of the non-T cells increased, in contrast to the decrease seen with the T-cell fraction. Although the number of experiments with non-T cells was few, our results indicate that it is essential to use T lymphocytes in this test system. Unsuccessful trials with the SCM method could in part be due to poor separation of T from non-T cells, which is obtained by iron-powder treatment and gradient centrifugation. The separation procedure of Cercek & Cercek (1977) gave inconsistent results in our hands. The procedure seems to be very sensitive, even for minor divergencies in the test condition, and the removal of responder cells from the top layer without admixture of non-responding cells was technically difficult. We had few technical problems using T lymphocytes which had been purified by rosetting. Cercek & Cercek (1977) have found up to 60–70% T lymphocytes in the responding cell fraction, and it is likely that the responding cells in their assay actually were T or mainly T lymphocytes. However, it still remains to show whether the B lymphocytes have a role in the SCM assay.

Sedimentation of T lymphocytes by sheep red cells, and shock treatment with distilled water did not seem to affect the ability of the cell to respond either to PHA or to cancer tissue.

The mean fluorescence polarization values of T lymphocytes, which were 0·198 ± 0·003 for healthy persons and 0·189 ± 0·005 for cancer patients compare well with the SCM values found by Cercek *et al.* (1974, 1975*b*, 1977). Also, the good differentiation between healthy persons and patients with non-malignant diseases on one hand, and cancer patients on the other agrees well with the observations of the other groups (Cercek & Cercek, 1977; Kreutzmann *et al.*, 1978; Pritchard *et al.*, 1978; Takaku *et al.*, 1977). The decrease of polarization after stimulation with PHA and cancer tissue was of the same order and similar to the reported findings.

In our study, the lymphocytes fro 7/10m colonic cancer patients which were tested against ovarian cancer tissue did not respond, as expected from the previous finding of Cercek & Cercek (1975*b*). However, in 3 other patients the T lymphocytes responded to ovarian cancer as well as to colonic cancer tissue. This could possibly mean that the 2 types of cancer had common antigenic sites, but these problems will need further evaluation.

In Case No. 15 colonic cancer was diagnosed when the test was performed, but later the diagnosis turned out to be pancreatic carcinoma. We found no response to colonic cancer tissue, but a 20% decrease of P after stimulation with tissue from cancer of the ovary. This patient was a man, and the response could possibly indicate another example of a cross-reaction.

The patient with ulcerative colitis, who showed no response either to colonic cancer tissue or to PHA, may represent a special premalignant group (Cercek &

Cercek, 1975*a*), although further work will be necessary to establish such criteria. The test was repeated some weeks afterwards with the same result. Clinically there was no evidence of malignancy. In the 2 other cases with non-malignant diseases, the response was similar to that found in apparently healthy persons, which is in good accordance with the observations of Cercek & Cercek (1977).

### CONCLUSIONS

Our observations indicate that the cells which respond in the SCM test are T lymphocytes. Our method for separation of lymphocytes differs from that reported by the Cerceks, but we have confirmed the observations that it may be possible by the SCM test to differentiate between persons with and without malignant disease, although further investigations are necessary to evaluate the clinical validity of the test. In our study, few patients with non-malignant disorders were included. It is important to do more extensive studies, particularly in patients with ulcerative colitis or in other diseases which possibly involve immune mechanisms, *i.e.* patients with irregular antibodies or antigen-antibody complexes.

The authors thank Drs Helge Bell, Rolf Grønvedt, Kjell Kjørstad and Rolf Kåresen for providing us with the cancer-tissue specimens and blood samples. We also thank Drs L. Cercek and B. Cercek for valuable advice.

### REFERENCES

Albrechtsen, D. & Lied, M. (1978) Stimulating capacity of human lymphoid cell subpopulations in mixed lymphocyte culture. *Scand. J. Immunol.*, **7**, 427.

Bagshawe, K. D. (1977) Workshop on macrophage electrophoretic mobility (MEM) and structuredness of cytoplasmic matrix (SCM) tests. *Br. J. Cancer*, **35**, 701.

Böyum, A. (1975) Isolation of lymphocytes, granulocytes and macrophages. *Scand. J. Immunol.*, **5** (Suppl. 5), 9.

Cercek, L., Cercek, B. & Franklin, C. I. V. (1974) Biophysical differentiation between lymphocytes from healthy donors, patients with malignant diseases and other disorders. *Br. J. Cancer*, **29**, 345.

Cercek, L. & Cercek, B. (1975*a*) Changes in the SCM response ratio ($RR_{SCM}$) after surgical removal of malignant tissue. *Br. J. Cancer*, **31**, 250.

Cercek, L. & Cercek, B. (1975*b*) Apparent tumour specificity with the SCM test. *Br. J. Cancer*, **31**, 252.

Cercek, L. & Cercek, B. (1977) Application of the phenomenon of changes in the structuredness of cytoplasmic matrix (SCM) in the diagnosis of malignant disorders: a review. *Eur. J. Cancer*, **13**, 903.

Kaplow, L. (1965) Simplified myeloperoxidase stain using benzidine dihydrochloride. *Blood*, **26**, 215.

Kreutzmann, H., Fliedner, T. M., Galla, H. J. & Sackmann, E. (1978) Fluorescence-polarization changes in mononuclear blood leucocytes after PHA incubation. Differences in cells from patients with and without neoplasia. *Br. J. Cancer*, **37**, 797·

Pritchard, J. A. V., Seaman, J. E., Evans, I. H. & 5 others (1978) Cancer-specific density changes in lymphocytes after stimulation with phytohaemagglutinin. *Lancet*, ii, 1275.

Takaku, F., Yamanaka, T. & Hashimoto, Y. (1977) Usefulness of the SCM test in the diagnosis of gastric cancer. *Br. J. Cancer*, **36**, 810.

Walford, R. L., Gallagher, R. & Troup, G. M. (1965) Human lymphocytes typing with isologous antisera; technical considerations and a preliminary study of the cytotoxic reaction system. *Transplantation*, **3**, 387.